# Genetic diversity and population structure of Ethiopian *Capsicum* germplasms

**DOI:** 10.1371/journal.pone.0216886

**Published:** 2019-05-21

**Authors:** Abate Mekonnen Solomon, Koeun Han, Joung-Ho Lee, Hea-Young Lee, Siyoung Jang, Byoung-Cheorl Kang

**Affiliations:** Department of Plant Science, Plant Genomics and Breeding Institute, Vegetable Breeding Research Center, College of Agriculture and Life Science, Seoul National University, Seoul, Korea; Chungnam National University, REPUBLIC OF KOREA

## Abstract

We established a collection of 142 *Capsicum* genotypes from different geographical areas of Ethiopia with the aim of capturing genetic diversity. Morphological traits and high-resolution melting analysis distinguished one *Capsicum baccatum*, nine *Capsicum frutescens* and 132 *Capsicum annuum* accessions in the collection. Measurement of plant growth parameters revealed variation between germplasms in parameters including plant height, stem thickness, internode length, number of side branches, fruit width, and fruit length. Broad-sense heritability was maximum for fruit weight, followed by length and width of leaves. We used genotyping by sequencing (GBS) to identify single-nucleotide polymorphisms (SNPs) in the panel of 142 *Capsicum* germplasms and found 2,831,791 genome-wide SNP markers. Among these, we selected 53,284 high-quality SNPs and used them to estimate the level of genetic diversity, population structure, and phylogenetic relationships. From model-based ancestry analysis, the phylogenetic tree and principal-coordinate analysis (PCoA), we identified two distinct genetic populations: one comprising 132 *C*. *annuum* accessions and the other comprising the nine *C*. *frutescens* accessions. GWAS analysis detected 509 SNP markers that were significantly associated with fruit-, stem- and leaf-related traits. This is the first comprehensive report of the analysis of genetic variation in Ethiopian *Capsicum* species involving a large number of accessions. The results will help breeders utilize the germplasm collection to improve existing commercial cultivars.

## Introduction

Members of the genus *Capsicum* in the Solanaceae family, commonly known as chilli peppers, are major crop plants and are almost cosmopolitan in distribution [1]. Chilli pepper fruits are used as spices, as vegetables and for medicinal purpose [2] and are a significant source of Vitamins A and C. They are also used as natural coloring agents, cosmetics and active ingredient in host defense repellents. Some are also used as ornamentals [3, 4]. The genus includes 27 species, of which five are known to be domesticated [5]. The five cultivated species of *Capsicum*, namely *C*. *annuum* L., *C*. *chinense* Jacq., *C*. *frutescens* L., *C*. *baccatum* L. and *C*. *pubescens* Ruiz et Pav., represent the most economically important vegetables worldwide [6].

The independent diffusion and subsequent domestication of the five cultivated species of *Capsicum* from its primary center of cultivation, Bolivia and Peru [2], to Europe by Columbus, and from there to Africa, India and China resulted in the crop's diversification due to human activity and in the development of non-deciduous, pendant, larger and non-pungent fruits with greater shape variation and increased fruit mass in several *Capsicum* growing areas [6]. Characterization of genetic diversity in various *Capsicum* species growing in different regions has been reported [7]. It has also been shown that ecological distribution has a significant influence on the genetic diversity of plants, including *Capsicum* [8].

According to Bozokalfa and Eşiyok [3], capsicums were introduced to Ethiopia first by the Portuguese in the 17^th^ century and subsequently from all over the world; they have since been cultivated for centuries and adapted to varied pocket agro-ecological zones. Such situations contributed to the evolution of local Ethiopian genotypes with different fruit types, pungency levels and disease resistance [9]. Adaptation and cultivation in the wide range of agro-ecologies of Ethiopia may have led to the evolution of accessions with great variation in many important traits. In this regard, an Ethiopian origin small fruited, pungent *C*. *annuum* inbred line, H3, was reported to be the most important and persistent source of powdery mildew (*Leveillula taurica*) and have been used to breed resistant varieties [10]. Different pepper cultivars have been produced and used as spice and vegetable crops ever since the first introduction of the genus. Pepper is consumed in many different forms in Ethiopia. The green fruit, known locally as “karia”, is eaten raw as a salad, and the dried red fruit is ground into powder and added to a sauce known as “wot”. Eating hot pepper is a deeply rooted Ethiopian food habit, and hot peppers are cultivated over more than 246,000 ha in Ethiopia [11]. Owing to its centrality in the daily diet of most Ethiopian societies, hot pepper plays an important role in the national economy. Though the number of local collections was small and investigations were based mainly on morphological assessment, with only limited use of genetic markers, previous work by Aklilu et al. [12], Shumbulo et al. [13], Marame et al. [14] and Geleta et al. [3] on Ethiopian *Capsicum* species indicated the existence of considerable genetic variability.

Germplasm diversity is crucial to successful breeding programs. Such diversity is important for broadening the genetic base, as it increases the probability of finding more unique genes for which two parents have different alleles (that is, the genetic distance). Numerous methods have been used to estimate genetic diversity among *Capsicum* genotypes, including multivariate analyses of large numbers of phenotypic descriptors [3, 7, 15] as well as of cytological [1], biochemical [16] and molecular variations [7, 17]. Since most morphological traits are polygenic and their expression depends on environmental factors, among others, the use of molecular markers is the most suitable method for estimating genetic diversity due to its ability to recognize specific DNA sequences in closely related genotypes, irrespective of growth stage, time, place and agronomic practices. Various types of DNA markers, such as restriction fragment length polymorphisms (RFLPs), random amplified polymorphic DNA (RAPDs), amplified fragment length polymorphism (AFLPs) and microsatellite repeats (or simple sequence repeats, SSRs), can be used to determine relationships and genetic variation levels in wild and domesticated crops, including *Capsicum* spp. [6]. Next-generation sequencing (NGS)-based genotyping methods have recently been used for whole-genome sequencing and for re-sequencing projects. In NGS, the genomes of several specimens are sequenced to discover large numbers of single-nucleotide polymorphisms (SNPs) that can be used to explore within-species diversity, construct haplotype maps and perform genome-wide association studies (GWAS) [18]. NGS has made routine screening of plant germplasm feasible and cost-effective [19]. The use of SNP markers provides the most useful information for detecting genetic diversity and determining genetic relationships between lines, owing to the abundance of SNPs in plants, to the technique's flexibility, low error rate, high speed of detection and cost-effectiveness and to the ease with which the resulting data can be converted to universal genotype information from different technological sources [4]. Genotyping by sequencing (GBS) is a genome-wide reduced representation of SNPs obtained using Illumina sequencing technology [18]. The use of restriction enzymes in GBS reduces genome complexity by avoiding the sequencing of repetitive regions, resulting in more straightforward bioinformatics analysis for large genomes [19, 20]. It is thus a rapid, high-throughput, genome-wide and cost-effective tool for SNP discovery [18]. It is helpful for genotyping without prior knowledge about the genome of the species and is useful for exploring plant genetic diversity on a genome-wide scale [4]. In the last few years, GBS has been used to investigate the genetic diversity of many crop species, including maize, rice, barley, tomato, wheat, sorghum, soybean, watermelon and *Capsicum* [21–28].

The present study was undertaken to characterize *Capsicum* germplasms collected from different localities of the six regions of Ethiopia using morphological and molecular markers to explore the genetic diversity available in a wide collection of germplasm. The data presented herein may be useful to understand the diversity of *Capsicum* in Ethiopia and use the information for the breeding purpose. Similarly, our finding may give additional insight into the quantitative trait loci controlling fruit weight.

## Methods

### Plant materials

**Fig 1:**
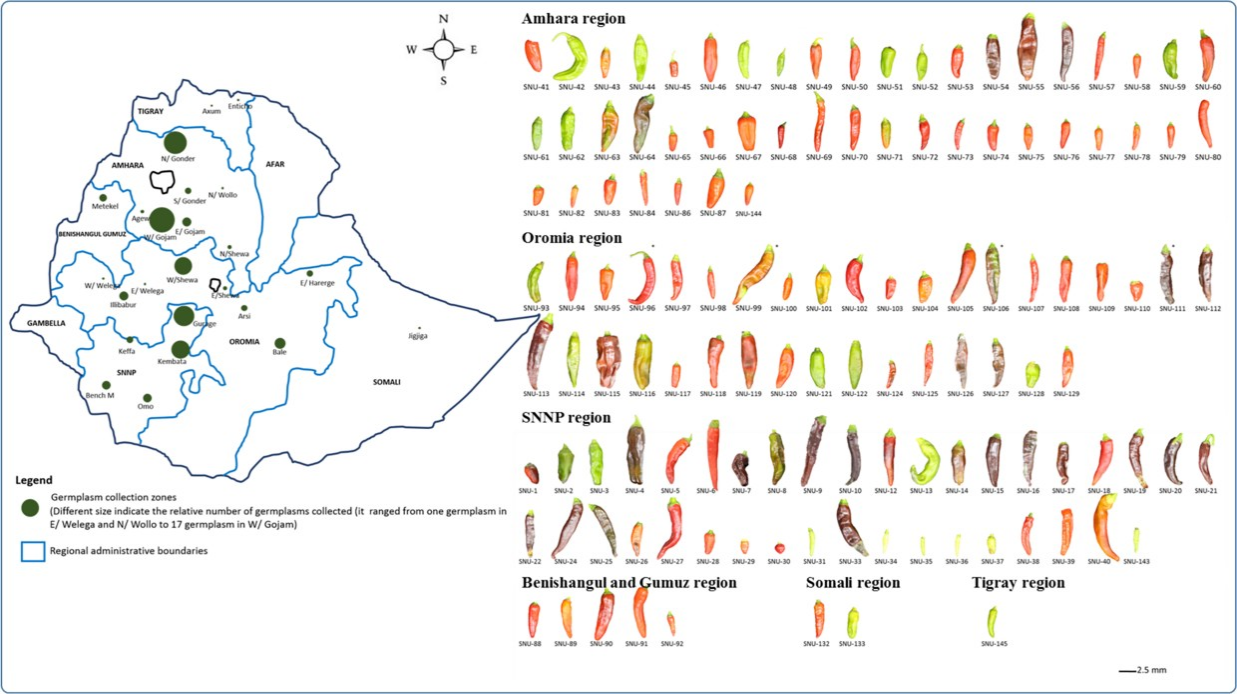
Map of germplasm collection areas of Ethiopian *Capsicum* collection regions. Germplasm origins were Amhara (47 germplasms); Benishangul Gumuz (5 germplasms); Oromia (37 germplasms); Southern Nations, Nationalities and Peoples (SNNPs) (40 germplasms); Somali (2 germplasms); Tigray (2 germplasms) and unknown (6 germplasms).

The germplasm collection of 142 genotypes used in this study was obtained from the Ethiopian Biodiversity Institute (EBI). These germplasms were collected from different pepper-growing areas of the country: 47 from eight zones of Amhara (11° 39′ 38.88″ N, 37° 57′ 28.08″ E); five from the Metekel zone of Benishangul Gumuz (10° 20′ 0″ N, 34° 40′ 0″ E); 38 from eight zones of Oromia (7° 59′ 20.62″ N, 39° 22′ 52.25″ E); 40 from five zones of the Southern Nations, Nationalities and Peoples (SNNPs) region (6° 3′ 31.03″ N, 36° 43′ 38.28″ E); one from the Jigjiga area of Somali (7° 26′ 19.43″ N, 44° 17′ 48.75″ E); and two from different weredas (districts) of Tigray (14° 8′ 11.68″ N, 38° 18′ 33.58″ E) (Fig 1). Ten germplasms used in this experiment have no accession passport data and are thought to be recent introductions. Among the germplasms, 42 were classified into particular species of *Capsicum* by the EBI based on the descriptor: four as *C*. *frutescens* and 38 as *C*. *annuum*. According to the germplasm descriptors, the EBI collected peppers in Ethiopia during 16 different years since the first germplasm record from the Limu wereda of the Oromia region in 1978. The majority of *Capsicum* germplasm collection (59%) was done between 1984 and 1990, during which most geographical areas with significant pepper cultivation were covered. In this experiment, three plants of each of the 142 germplasms were grown under greenhouse conditions at Biotong Seed Co. Ltd., Anseong, Republic of Korea, in 2017. Seeds were disinfected using 2% sodium chlorate and 10% trisodium phosphate.

### Morphological characterization and statistical analysis

For each germplasm, 12 growth traits and nine flower-, 17 fruit- and two seed-related traits were evaluated according to the descriptions used by the Rural Development Administration (RDA) gene bank, South Korea, with some modifications (S1 Table). Morphological traits such as plant type (PT), plant height (PH), plant width (PW), main stem length (MSL), internode length (INL), number of side branches (NSB), stem thickness (ST), stem color (SC), leaf color (LC), and leaf length and width (LL and LW) were recorded 114 days after sowing. One representative flower from each plant was assessed for stamen number (SN), filament color (FC), anther color (AC), petal color (PC), petal length (PL), petal width (PW), petal number (PN) and calyx shape (CS) (S1 Fig).

Tomato analyzer version 3.0 was used to measure fruit perimeter (FP), fruit area (FA), fruit width mid height (FWMH), maximum fruit width (MFW), fruit height mid-width (FHMW) and fruit curved height (FCH) as previously described [29]. A Spearman’s rank correlation coefficient was calculated among all the variables, including the altitude at which the germplasms were collected. Kaiser-Meyer-Olkin (KMO) and Bartlett’s tests were performed using SPSS software 22.0 to measure sampling adequacy and sphericity, respectively [30] (S1 Table).

The phenotypic (PCV) and genotypic (GCV) coefficients of variation were estimated as percentages of the corresponding phenotypic and genotypic standard deviations from the trait grand means as used by Khan et al. [31]. Estimates of broad-sense heritability in percent were obtained using the formula suggested by Burthon and de Vane [32].

### HRM-PCR amplification and data analysis

To the identify the species of 142 *Capsicum* germplasms, high-resolution melting (HRM) was performed as described previously [15, 33]. A Rotor-Gene 6000 real-time PCR thermocycler (Corbett Research, Sydney, Australia) was used with the following PCR amplification conditions: 95°C for 10 min; 50 cycles of 94°C for 20 s, 55°C for 20 s, and 72°C for 40 s; 95°C for 60 s; and 40°C for 60 s. For HRM analysis, an increase of 0.1°C temperature per minute from 65°C to 90°C was used. A combination of five markers (S5 Table) developed previously was implemented for species identification [15]. Five reference materials, viz. *C*. *annuum*, *C*. *chinense*, *C*. *frutescens*, *C*. *chacoense* and *C*. *baccatum*, were used.

### DNA extraction and library construction for genotyping by sequencing

Two or three young leaves from each germplasm were used as sources of DNA. Total DNA was extracted using the modified cetyl trimethylammonium bromide (CTAB) method as described previously [34]. The concentration and purity of DNA samples were determined with a NanoDrop 1000 spectrophotometer (NanoDrop Technologies, Wilmington, DE, USA). DNA samples with absorbance ratios above 8 at 260/280 nm were used for analysis [15], and gel electrophoresis was conducted on a 0.8% agarose gel.

Two GBS libraries were constructed based on a modified protocol as used previously [19, 35] using a two-enzyme system, *Pst*I (rare cutter) and *Mse*I (frequent cutter). Each GBS library was a 96-plex library consisting of 60 and 82 samples from the *Capsicum* diversity panel (S2 Table).

### Sequencing data analysis, SNP identification and genome-wide association analysis

Sequencing was performed with an Illumina HiSeq 2500 (Macrogen Inc., Seoul, Korea). Data analysis and SNP identification were performed as described previously [36]. Raw reads were de-multiplexed in accordance with individual barcodes, and the adapter and barcode sequences were removed using commercially available CLC genomic workbench software (version 6.5). Trimmed reads were mapped to CM334 chromosome version 1.6 (Pepper.v.1.6.total.chr.fa) by Burrows-Wheeler Aligner (BWA) [37]. The SAMtools program was used to group and sort the reads by chromosomal order [38]. The Genome Analysis Toolkit (GATK) program was used to call SNPs over whole chromosomes [39]. From the 142 total germplasms, 12 were omitted from SNP-based analysis due to significant loss of reads.

The 53,284 filtered SNPs were used for genome-wide association (GWAS) mapping. The default settings of Genomic Association and Prediction Integrated Tool of the R package were used to estimate GWAS based on the compressed mixed linear model [40]. SNPs with a calling rate of more than 0.1 were retained and FILLIN in TASSEL was used for imputation. R^2^ values and imputed ratio of minor and major alleles were used to select suitable imputed quality. A final filtering was performed based on minor allele frequency of more than 0.05, SNP coverage >0.6 and inbreeding coefficient >0.8. The *P* values of SNPs from GWAS were subjected to a false-discovery rate (FDR) analysis, and Bonferroni correction was done to reduce false-negative results from the GWAS analysis. A significance threshold level at a *P* value of 0.05 was set after Bonferroni multiple-test correction.

### Genetic diversity and population structure analysis

For each SNP, polymorphic information content (PIC), heterozygosity (H2), gene diversity, genotype number, allele number and allele frequency were calculated using Power Marker software [17], and the genetic diversity for the entire set of *Capsicum* genotypes as well as the geographically based subpopulations were also identified by PowerMaker version 3.25. To investigate the population structure, assess genetic diversity and remove near-duplicates (i.e., highly similar genotypes), both parametric and non-parametric approaches were used. Pairwise geographic distances between accessions, pairwise *F*_ST_ between accessions in the different groups and analysis of molecular variances (AMOVAs) were calculated using GenAlEx 6.503, with 999 permutations for testing variance components [41].

Population structure was estimated from 13,998 selected SNPs from the 53,284 polymorphic SNPs used in GWAS analysis that can easily be handled by the software used for population structure analysis. Population structure was determined using STRUCTURE software (http://pritch.bsd.uchicago.edu/structure.html) [42], which was run from the command line using the admixture model, a burn-in period length of 10,000 and 10,000 Markov-chain Monte Carlo (MCMC) iterations after burn-in. Ten independent runs were performed for each *K* from *K* = 1 to *K* = 5. The best number of *K* was chosen with the DeltaK method [24] by running the STRUCTURE HARVESTER software [43].

### Phylogenetic and principal-coordinate analyses

Phylogenetic trees were produced using genotyping data with 53,284 SNP markers using both the unweighted neighbor-joining method and the hierarchical cladding method based on the dissimilarity matrix calculated with Manhattan index, as implemented in the DARwin software (version 6.0.9) [44], and was visualized with Dendroscope (version 3.5.9) [45]. Inkscape 0.92 was used to make annotations and to apply visual effects to the phylogenetic tree. Principal-coordinate analyses (PCoA) were performed with GenAlEx version 6.503 [41].

## Results

### Species identification based on HRM genotyping

**Fig 2:**
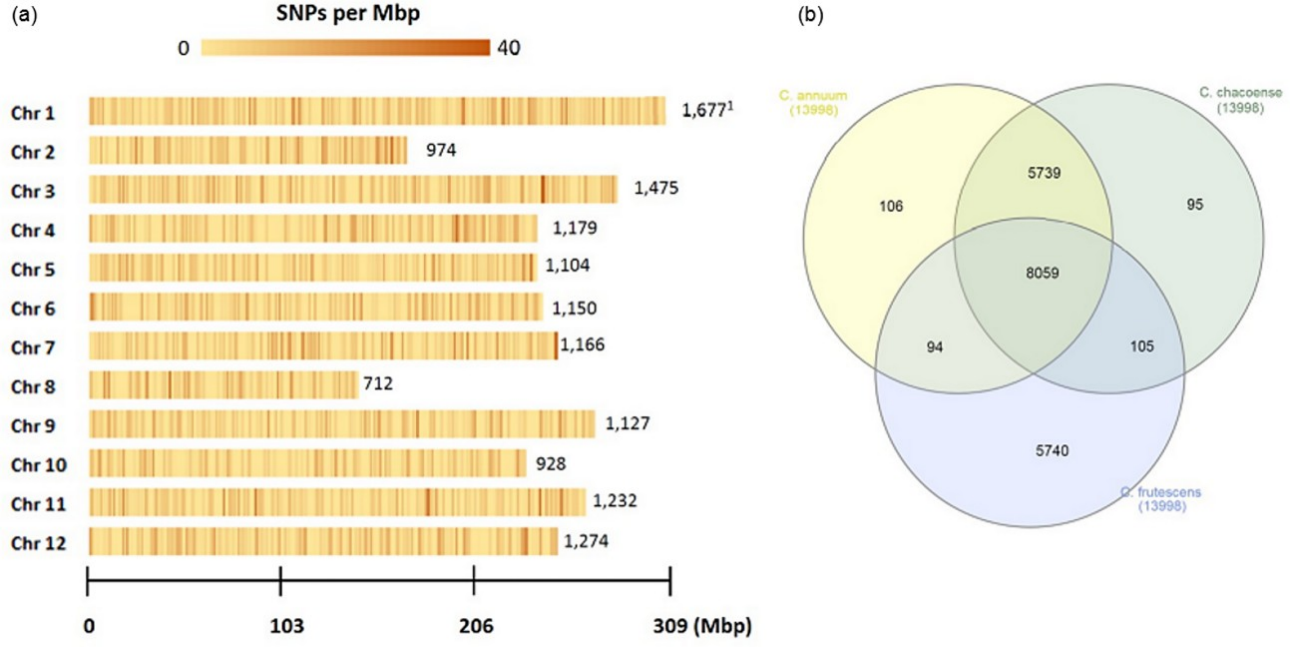
Single-nucleotide polymorphism (SNP) density and distribution across the 12 chromosomes of the *Capsicum* germplasms. (A) SNP density (number of SNPs per Mbp) of Ethiopian *Capsicum* germplasms digested using *Pst*I and *Mse*I. (B) Venn diagram of unique and shared SNPs of *Capsicum* germplasms, kept in each dataset after SNP filtering. The figure was drawn using the interactiVenn website.

In addition to the preexisting identification of some of the germplasms by EBI, and the assessment of morphological features [15] and SNP information (Figs 1 and 2 and S1 Table), we obtained further confirmation of species through a real-time HRM-PCR protocol as described earlier [33]. We used five high-resolution melting markers (HRMs) to assign germplasms to different species (S5 Table) [15]. The 142 germplasms were classified into three species, *C*. *annuum*, *C*. *frutescens* and *C*. *baccatum*. The four markers C2_At5g19560, C2_At5g50020, Waxy and PepTrn showed a polymorphism that was highly specific for the three species (S2 Fig). Eighty-six *C*. *annuum* accessions were identified by the C2_At5g50020 marker, of which 12 were further confirmed by both Waxy and PepTrn, 28 by Waxy and 33 by PepTrn. The remaining 46 *C*. *annuum* accessions were identified by the single markers C2_At5g19560 (two), C2_At5g50020 (13), Waxy (eight) and PepTrn (32). Confirmation of *C*. *baccatum* status was made on the basis of the specific melting curve shape using PepTrn and C2_At5g50020 markers. *C*. *frutescen*s accessions were likewise identified on the basis of melting curve shape using the markers Waxy and PepTrn. C2_At5g50020 also identified two *C*. *frutescens* accessions (SNU-142 and SNU-143).

### Qualitative and quantitative morphological characterization

We evaluated the qualitative properties of plants, flowers, leaves and fruits based on descriptions used by Rural Development Administration (RDA) gene bank of South Korea. Plant habits were spreading (12.9%), half-spreading (59.7%), erect (26.6%) and fasciculate 0.7%). Spreading types were collected from Amhara, Oromia and SNNPs at altitudes ranging between 1000 and 2570 meters above sea level (m.a.s.l.), while the altitude range for half-spreading plant types was 1150–2780 m.a.s.l. distributed in different locations of Amhara, Benishangul Gumuz, Oromia, SNNPs, Somalia and Tigray. A relatively narrower altitude range (1200–2060 m.a.s.l.) was observed for the erect *Capsicum* types from Amhara, Oromia, SNNPs and Tigray. The only fasciculate *Capsicum* type was obtained from the Bibugn wereda of Amhara at an altitude of 1850 m.a.s.l. (S1 Table).

Measurement of plant growth parameters revealed variation between germplasms. Plant height (48–175 cm), stem thickness (6–33 mm), internode length (4–18.5 cm) and number of side branches (4–30) all evidenced variation. The seven tallest germplasms (140–175 cm in height) were collected from SNNPs at a mean altitude of 1300 m.a.s.l. Short to medium-height germplasms were collected from all *Capsicum* growing regions, with a wider altitude range (1000–2570 m.a.s.l.). Wider variation in stem thickness was observed in *C*. *annuum* (6.32–33 mm) than in *C*. *frutescens* (7.2–15.7 mm). Mean stem thickness for *C*. *baccatum* was 23.3 mm. The maximum mean internode length (11.4 cm) was measured for *C*. *frutescens*, while the minimum (9.23 cm) average internode length was observed for *C*. *annuum*. Similarly, the maximum average number of side branches below first node (17, with a range of 12–21) was counted in *C*. *frutescens*, followed by *C*. *annuum* (15, with a range of 4–30) (S7 Table, S1 Table and S3 Fig).

Other morphological traits similarly showed variation (Fig 1 and S1 Fig). Most germplasms (97.2%) had one flower per axil, while the remaining four germplasms, which were collected from Amhara and SNNPs (SNU17-2, SNU17-13 and SNU17-76 from *C*. *annuum* and SNU17-34 from *C*. *frutescens*), had two. The maximum measured fruit length was 14.4 cm and the minimum was 1.8 cm, each found for one accession. Respectively, 24%, 22%, 21%, 19%, 18% and 12% of germplasms had fruit lengths measuring 8, 5, 4, 7, 6 and 9 cm. The minimum fruit length score was 3 cm (seen for seven germplasms). Fruit width ranged between 0.3 and 3.2 cm. The majority of germplasms (39%) had fruit widths measuring 2 cm, with 35% measuring 1.5 and 22% measuring 2.5 cm. The fruit shape index, which is the ratio of fruit length to width, ranged between 1.5 and 13. Whereas the fruits of 73.5% of germplasms have two locules, 24.3% had three locules. Only three germplasms (SNU17-67, SNU17-133 and SNU17-140) displayed four locules. Weights of fruits varied between 1 and 22 g. Seed number per fruit ranged from zero for one germplasm (SNU17-40, *C*. *annuum*) to over 175 for two other germplasms of *C*. *annuum* (SNU17-102 and SNU17-116).

Estimates of phenotypic (PCVs) and genotypic coefficients of variation (GCVs), broad-sense heritability and genetic advance are shown in Table 1. Across the traits studied, the PCV values ranged from 39.6% for plant width to 99.6% for fruit weight. Similar to the latter, PCV values were high for number of seeds per fruit, fruit length, fruit width, pericarp thickness, fruit perimeter, internode length, leaf length and main stem length, with respective values of 81.5%, 65.6%, 58.7%, 58.3%, 53.3%, 53.0%, 52.4% and 50.6%. In contrast, number of side branches, leaf length, pedicel length, plant height, stem thickness and plant width showed comparatively lower PCV values (<50%). The GCV estimates were lowest (31.9%) for plant width and highest (98.6%) for fruit weight. High GCV values were also recorded for seeds per fruit, leaf width and fruit length. However, relatively low GCV values (<50%) were recorded for fruit width, pericarp thickness, fruit perimeter, internode length, number of side branch, leaf length, main stem length, pedicel length, plant height and stem thickness. Broad-sense heritability was greatest for fruit weight (98.1%), followed by length and width of leaves with respective values of 77.7% and 77.1%. The heritability values of the remaining traits ranged from 62.7 to 75.9%. Genetic advance (GA) as percentage of the mean ranged from 1.7% to 107.7% for plant height and pericarp thickness, respectively (Table 1).

**Table 1:** Estimation of genetic parameters of different traits.

Characters	Range	Mean ± SE	CV	Genetic variance	Phenotypic variance	Grand mean	Heritability (%)	GCV (%)	PCV (%)	Genetic advance (%)
Plant height	43–175	100.83 ± 1.29	21.4	1205.7	1672.0	100.8	72.1	34.4	40.6	1.74
Plant width	26.67–90.67	62 ± 0.8	23.5	391.2	604.2	62.0	64.8	31.9	39.6	2.68
Main stem length	17.67–64	37.42 ± 0.61	29.4	237.2	357.9	37.4	66.3	41.2	50.6	4.49
Internode length	3.67–18.17	9.32 ± 0.16	28.9	17.1	24.4	9.3	70.1	44.4	53.0	18.52
Number of side branch	4–23.7	14.61 ± 0.22	24.2	39.5	52.1	14.6	75.9	43.0	49.4	12.31
Stem thickness	5.83–24.37	13.76 ± 0.18	22.6	21.4	31.0	13.8	68.8	33.6	40.5	12.44
Leaf length	8.33–136.67	84.4 ± 1.28	23.4	1307.9	1696.4	84.4	77.1	42.9	48.8	2.15
Leaf width	4.2–80	41.59 ± 0.67	24.8	368.9	474.8	41.6	77.7	64.2	52.4	4.37
Pedicel length	1.03–6.23	3.52 ± 0.05	27.4	1.7	2.6	3.5	64.4	36.8	45.8	47.03
Fruit length	1.53–10.93	6.42 ± 0.13	34.6	12.8	17.8	6.4	72.2	55.8	65.6	27.30
Fruit width	0.27–3.53	1.71 ± 0.03	31.7	0.72	1.01	1.7	70.9	49.4	58.7	101.34
Fruit weight	0.73–15.4	6.8 ± 0.18	13.6	39.59	40.34	6.8	98.1	98.6	99.6	2.04
Pericarp thickness	0.3–3.23	1.59 ± 0.03	32.7	0.59	0.86	1.6	68.6	48.3	58.3	107.65
Seed number/fruit	12–167.67	78.98 ± 2.12	49.7	2596.55	4138.6	79.0	62.7	64.5	81.5	2.07
Fruit perimeter	2.71–19.94	12.37 ± 0.21	28.7	30.8	43.5	12.4	70.9	44.9	53.3	14.04

### GBS and single-nucleotide polymorphisms

We identified 2,831,791 genome-wide SNPs in our germplasm panel. We filtered these by removing rare alleles (with prevalence less than 5%), alleles with high missing ratios (absent from more than 30% of the germplasms) and alleles with high heterozygosity (more than 80%). To explore the genetic diversity of the panel, we analyzed all the germplasms using 53,284 high-quality SNPs. The chromosomal distribution and proportion of polymorphic markers used for the competition is shown in S6 Table. We mapped the SNP density (number of SNPs per Mbp) and their distribution across the 12 chromosomes (Fig 2) and found that SNP densities varied across chromosomes. There was relatively high uniformity on chromosomes 3, 4, 5, 6, 8, 9, 10 and 12. On chromosome 2, a higher SNP distribution was found towards one end, and it was higher on both arms of chromosome 11. Relatively more SNPs were also recorded around the middle and one end of chromosome 7.

### Genetic diversity

The amount and organization of genetic diversity among the model-based populations is presented in Table 2. A high allele number was recorded in C2 (5.00), and the mean major allele frequency was greater in C1 (0.30) than in C2 (0.28). The average expected heterozygosity (a measure of genetic diversity), observed heterozygosity and polymorphic information content (PIC; denoting allelic diversity and frequency) values were 0.74, 0.02 and 0.69 for C1 and 0.75, 0.03 and 0.70 for C2, respectively. The PIC value was 0.692 in 35 *C*. *annuum* and 0.701 in six *C*. *frutescens* germplasms. Categorically, the average *C*. *annuum* PIC value for the major growing region (SNNPs) was 0.693 per marker, with a range running from 0.692 in six germplasms collected from Gurage and Kembata zones to 0.693 in 24 germplasms collected from Gurage, Kembata, Bench Maji, Keficho and Semen Omo zones. A similar trend was observed in the PIC values of *C*. *annuum* for the Amhara and Oromia regions, representing respectively 39 and 24 germplasms. For the *C*. *frutescens* germplasms of SNNPs, the average PIC value was 0.701 per marker, and for Oromia it was 0.964. The overall gene diversity and mean heterozygosity were 0.74 and 0.02, respectively.

**Table 2:** Genetic diversity analysis of *Capsicum* germplasms.

Subpop.	*N*	AN	MAF	*H*_e_	*H*_o_	PIC
C1	121	4.46	0.30 (0.29–0.30)	0.74 (0.740–0.744)	0.02 (0.004–0.104)	0.69 (0.69–0.70)
C2	9	5.00	0.28 (0.28–0.30)	0.75 (0.74–0.75)	0.03 (0.01–0.06)	0.70 (0.69–0.70)
Total	130	4.50	0.3 (0.28–0.30)	0.74 (0.74–0.75)	0.02 (0.004–0.104)	0.69 (0.69–0.70)

*N*, number of germplasms; AN, number of allele per locus; *H*_e_, expected heterozygosity (gene diversity); *H*_o_, observed heterozygosity; PIC, polymorphism information content.

### Analysis of molecular variance

To quantify the genetic diversity within and among subpopulations, we partitioned the total molecular variance into two clades according to the STRUCTURE simulation result. Averaged across the 130 germplasms, 92% of the total genetic diversity was partitioned between germplasms within the subpopulations, and only 8% was attributed to differences at the individual level (Table 3).

**Table 3:** AMOVA for the two subpopulations suggested by STRUCTURE for all Ethiopian *Capsicum* germplasms.

Source	Df	SS	MS	Var.	%
Among subpopulations	1	0.027	0.027	0.000	0%
Among germplasms	128	11.915	0.093	0.045	92%
Within germplasms	130	0.500	0.004	0.004	8%
Total	259	12.442		0.048	100%

*P* < 0.001. Df, degrees of freedom; SS, sum of squares; MS, mean square; Var., estimated variation.

### Population structure

**Fig 3:**
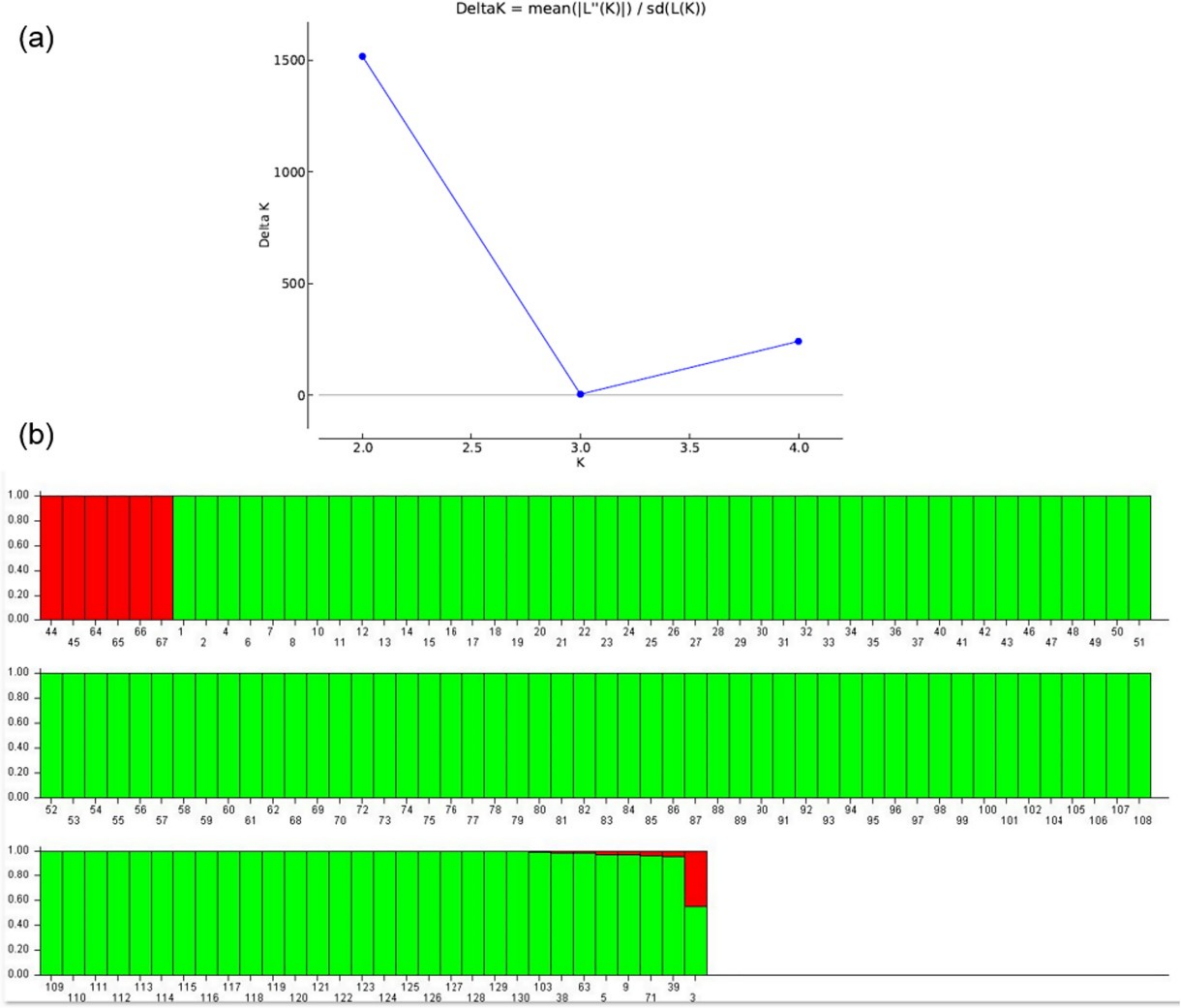
Population structure of 139 *Capsicum* germplasms. (a) STRUCTURE estimation of the number of subgroups from *K* values ranging from 1 to 10, by delta *K (*Δ*K)* values. (b) Population structure from *K* = 2. The two colors each represent one subpopulation (red, C1; green, C2), and the lengths of the colored segments shows the estimated membership proportion of each germplasm in the designated group.

We inferred the population structure of the 130 Ethiopian *Capsicum* germplasms using the program STRUCTURE 2.3.4 [46]. We carried out admixture model-based simulations by varying *K* from 1 to 5 with 10 iterations using 130 germplasms. The estimated likelihood (ln*P*(*D*)) was greatest for *K* = 2 (Fig 3), suggesting the presence of two main populations in the *Capsicum* germplasm panel [43]. The classification of germplasms into populations based on the model-based structure from STRUCTURE 2.3.4 (Fig 3 and S3 Table) showed that subpopulation C1 comprised 123 germplasms and subpopulation C2 comprised the remaining 7 germplasms. We tested the genetic variation within the subpopulations using the fixation index (*F*_st_) statistic for genetic differentiation. We observed a low average distance (*H*_E_) between individuals in the same clade in subpopulation C1 (0.05) and a higher *H*_E_ in subpopulation C2 (0.07). The *F*_ST_ values for subpopulations C1 and C2 were 0.713 and 0.850, respectively.

### Molecular phylogenetic and principal-coordinate analysis

**Fig 4:**
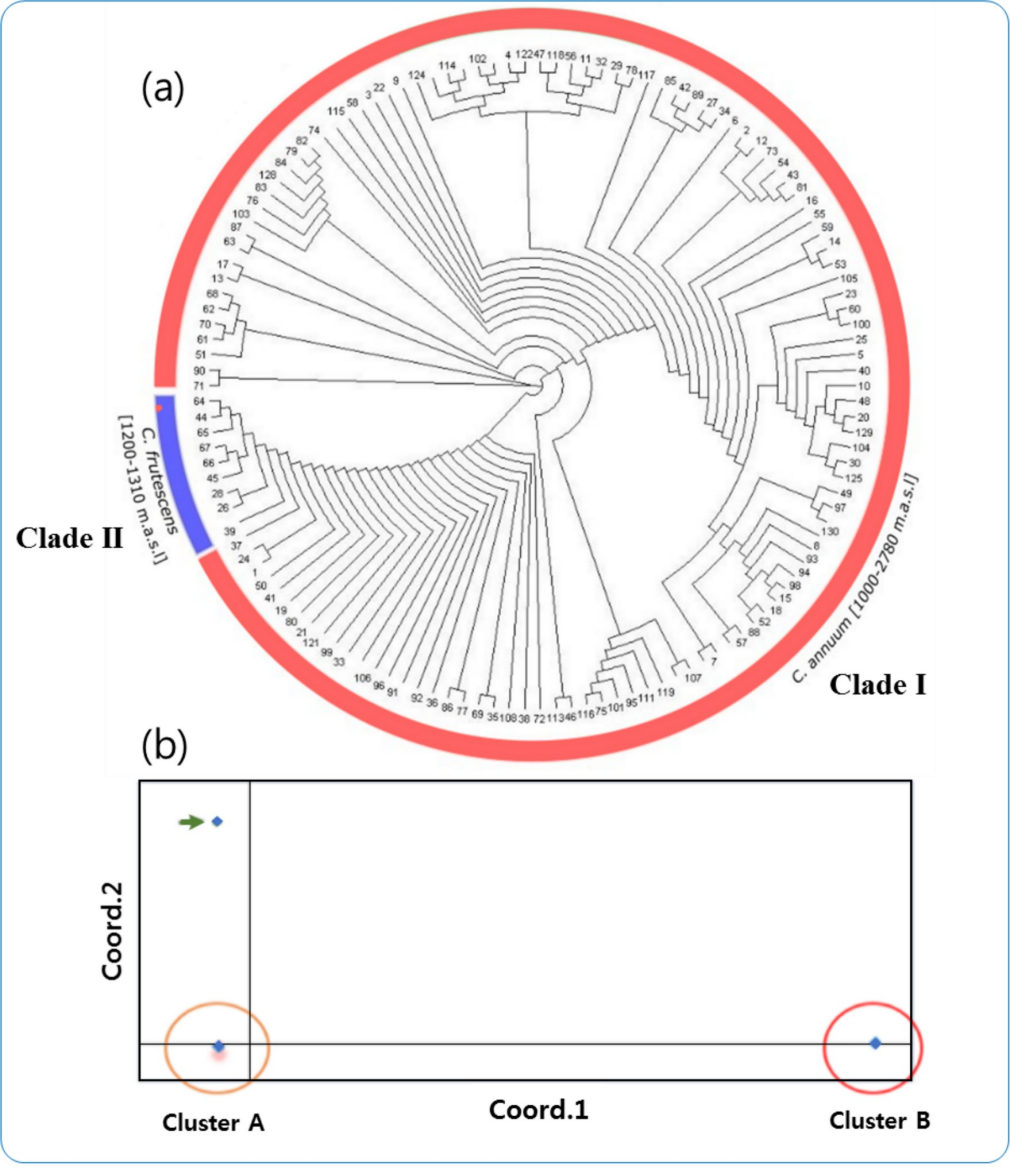
Phylogenic tree and principal-coordinate analysis results confirming the presence of two groups. (a) Unrooted neighbor-joining (NJ) tree of the 142 *Capsicum* germplasms. (b) Principal-coordinate analysis results showing diversity of *Capsicum* species clustered in the two subpopulations.

The unrooted phylogenetic tree with two clades is consistent with the model-based population structure, in which *C*. *frutescens* germplasms were grouped separately from *C*. *annuum* (Fig 4). Clade 1 contained *C*. *annuum* accessions growing in an altitude range between 1000 and 2780 m.a.s.l. and consisting mainly of germplasms collected from different growing localities of the SNNPs region (35 germplasms), Amhara (47 germplasms), Oromia (37 germplasms), Benishangul (5 germplasms), Somali (1 germplasm) and Tigray (2 germplasms), which accounted for 24%, 33%, 26%, 3.5%, 0.7% and 1.4% of germplasms, respectively. The growing altitude range of *C*. *frutescens* (1200–1310 m.a.s.l.) was narrow compared with that of *C*. *annuum* (Fig 4).

We also performed PCoA on 130 germplasms (Fig 4). This analysis largely supported the separation of the germplasms into two subpopulations fairly well distributed on the axes, with one variation as indicated by an arrow. Cluster A consisted mainly of *C*. *annuum*, a pattern also evidenced in the model-based genetic clustering using STRUCTURE and the phylogenetic tree. Germplasms in Cluster B were all from *C*. *frutescens*.

### GWAS for selected traits

**Fig 5:**
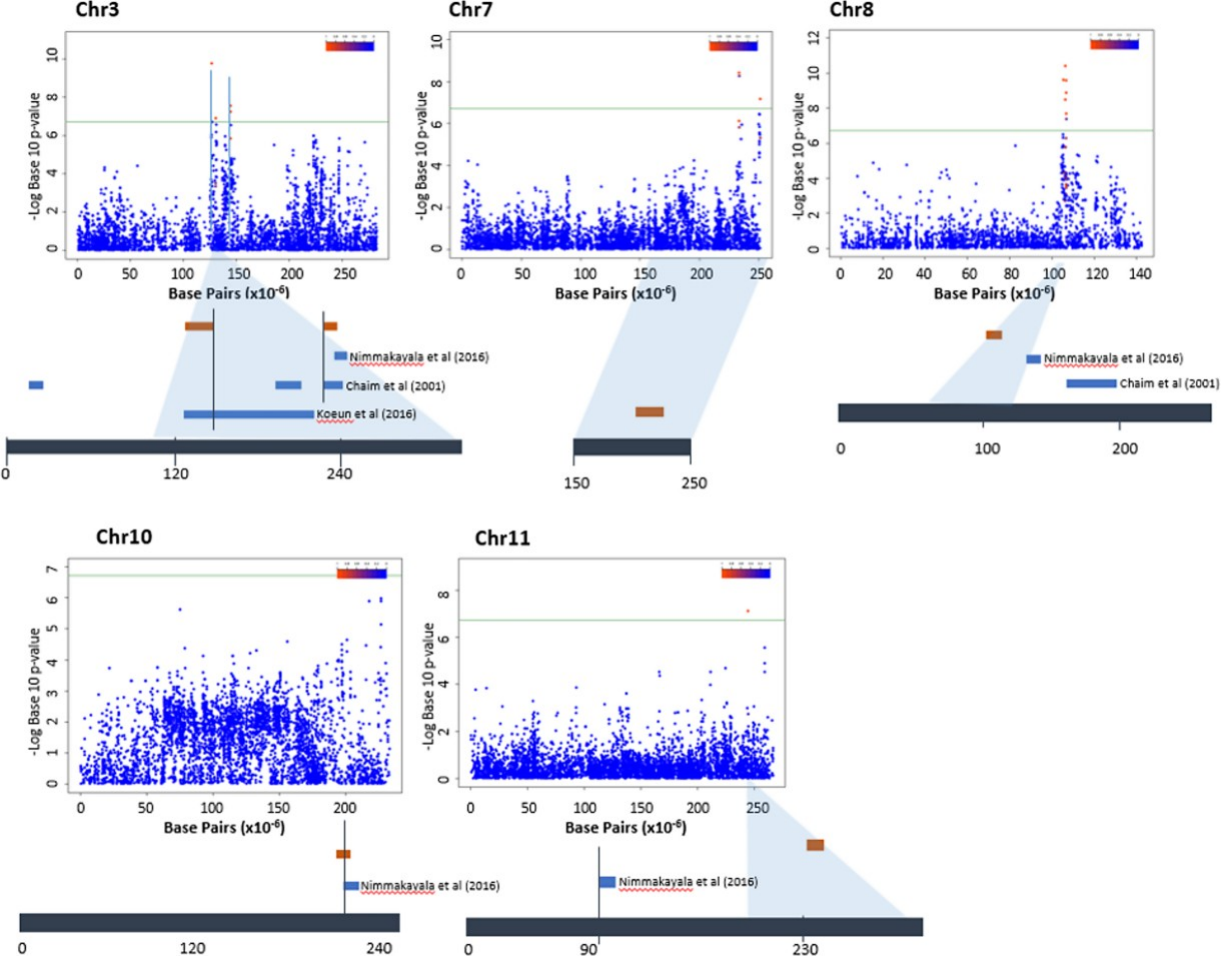
Manhattan plots of the genome-wide association study for fruit weight. Chromosome coordinates are displayed along the *X* axis with the–log_10_ of the association *P* value for each single nucleotide polymorphism displayed on the *Y* axis. A greater–log_10_ indicates stronger association with the trait. The green line denotes the significance threshold. The threshold for–log_10_(*P*) was 6.0. Blue thick horizontal bars denote QTL positions reported previously, while brown horizontal thick bars are our results.

Genome-wide association result on fruit weight is summarized by Manhattan plots in Fig 5. With a Bonferroni correction threshold of 5% (–log_10_(*P* > 6.03), the number of markers linked to various traits varied from a maximum of 187 for fruit length to a minimum of one for fruit shape index, leaf color, petal length, petal width and stem color (S8 Table). A total of 509 significant SNPs were identified, 81.53% of which were for fruit traits, 10.61% for leaf traits, 0.39% for petal length and width and 7.47% for stem-related traits. The largest fraction of significant SNPs (26.68%) was detected on chromosome 3, followed by chromosomes 8 and 9 with 16.11% and 13.56%, respectively. The smallest concentration of significant SNP markers was observed on chromosome 12 for fruit length, fruit number, fruit weight and petal width. SNP markers related to fruit traits (area, color, length, width, number, weight, shape-index, number of locules, pericarp thickness and perimeter) were distributed across all 12 chromosomes. While SNP markers for stem-related traits (internode length, hairiness, thickness, color and branching) are distributed on chromosomes 1, 2, 3, 4, 6, 9, 10 and 11, those for leaf-related traits (length, width and color) are localized on chromosomes 2, 3, 6, 7 and 9 (S4 Fig).

For fruit weight (Fig 5), two regions containing 12 SNPs were detected on chromosome 3, from 126.3 to 144.9 Mbps and from 223.02 to 223.04 Mbps, and one region was detected on chromosome 7 with seven SNPs in the region between 233.4 and 251 Mbps. There were ten SNP marker positions on chromosome 8 between 106.28 and 106.6 Mbps, and one each on chromosomes 10 and 11 at 226.8 and 244.4 Mbps, respectively. From this result, our research demonstrated the existence of three new regions of significant SNP markers for fruit weight on chromosomes 7, 10 and 11. A total of 27 protein-coding genes were predicted within the significant SNP regions based on the annotation of the CM334.v.1.6. genome (Table 4).

**Table 4:** List of 27 candidate genes located in chromosomes 3, 7, 8, 10 and 11 for the fruit weight.

SNP ID	Chromosome	Position	maf	P.value	Predicted protein	GO Name	InterPro GO Names
S03_126637882	3	126637882	0.27	1.76E-10	S-locus-specific glycoprotein S6	F:binding; F:electron transfer activity; C:membrane	no IPS match
S03_144571942	3	144571942	0.25	5.60E-08	hypothetical protein BC332_25369		no IPS match
S03_130472754	3	130472754	0.22	1.26E-07	DNA/RNA polymerases superfamily protein	F:binding; P:DNA metabolic process	no GO terms
S03_127229794	3	127229794	0.26	1.94E-07	Lysine-specific demethylase	P:phosphatidylserine biosynthetic process; C:integral component of membrane; F:transferase activity	no GO terms
S03_131045471	3	131045471	0.26	2.71E-07	hypothetical protein T459_16762	F:mannosyl-oligosaccharide 1,2-alpha-mannosidase activity; F:calcium ion binding; C:membrane	no GO terms
S03_144926307	3	144926307	0.24	2.90E-07	Putative gag-pol polyprotein, identical	F:nucleic acid binding; F:catalytic activity; F:zinc ion binding; F:1-deoxy-D-xylulose-5-phosphate synthase activity; P:DNA integration; P:terpenoid biosynthetic process	no IPS match
S03_130816018	3	130816018	0.22	6.81E-07	PREDICTED: uncharacterized protein LOC107844328	F:ion binding; F:organic cyclic compound binding; F:heterocyclic compound binding	no GO terms
S03_126408053	3	126408053	0.27	9.28E-07	Lysine-specific demethylase	F:nucleic acid binding; P:gene expression; F:transferase activity; P:cellular nitrogen compound metabolic process; P:cellular protein metabolic process	no GO terms
S03_223037785	3	223037785	0.48	1.05E-06	Malate dehydrogenase, glyoxysomal	F:catalytic activity	no IPS match
S03_223037803	3	223037803	0.48	1.05E-06	Malate dehydrogenase, glyoxysomal	F:catalytic activity	no IPS match
S03_139442074	3	139442074	0.26	1.11E-06	ATP-dependent DNA helicase SRS2-like protein	C:integral component of membrane; P:oxidation-reduction process	no IPS match
S07_233424834	7	233424834	0.15	3.94E-09	ATPase ASNA1 -like protein	F:protein serine/threonine kinase activity; F:ATP binding; C:endoplasmic reticulum; P:generation of precursor metabolites and energy; P:protein phosphorylation; P:proteolysis; P:transport; F:cysteine-type peptidase activity; C:integral component of membrane; F:ATPase activity; P:protein insertion into ER membrane; P:negative regulation of transcription, DNA-templated	no GO terms
S07_233499205	7	233499205	0.19	5.50E-09	Potassium transporter 5	P:cellular protein modification process; P:phosphate-containing compound metabolic process; F:methyltransferase activity; C:membrane; F:hydrolase activity; P:methylation; F:adenyl ribonucleotide binding; F:anion binding; C:intracellular part; F:catalytic activity, acting on a protein	no IPS match
S07_251125166	7	251125166	0.37	6.81E-08	putative copia-type protein	C:retrotransposon nucleocapsid; F:nucleic acid binding; F:motor activity; F:protein tyrosine phosphatase activity; F:ubiquitin-protein transferase activity; F:ATP binding; P:actin filament organization; F:protein tyrosine/serine/threonine phosphatase activity; P:DNA integration; C:integral component of membrane; C:myosin complex; P:protein ubiquitination; P:peptidyl-tyrosine dephosphorylation; F:ADP binding; F:actin filament binding	C:retrotransposon nucleocapsid
S07_250424475	7	250424475	0.29	3.48E-07	uncharacterized protein LOC114075241	F:nucleic acid binding; F:RNA-DNA hybrid ribonuclease activity; P:DNA integration; P:RNA phosphodiester bond hydrolysis, endonucleolytic	F:nucleic acid binding; F:RNA-DNA hybrid ribonuclease activity; P:DNA integration
S07_250605815	7	250605815	0.38	3.75E-07	TPR repeat-containing thioredoxin TDX	C:cell; F:transferase activity	no IPS match
S07_233424790	7	233424790	0.16	7.40E-07	ATPase ASNA1 -like protein	F:protein serine/threonine kinase activity; F:ATP binding; C:endoplasmic reticulum; P:generation of precursor metabolites and energy; P:protein phosphorylation; P:protein dephosphorylation; P:proteolysis; F:protein tyrosine/serine/threonine phosphatase activity; F:cysteine-type peptidase activity; C:integral component of membrane; F:ATPase activity; P:protein insertion into ER membrane; P:negative regulation of transcription, DNA-templated	no GO terms
S07_249749737	7	249749737	0.33	1.07E-06	ADP,ATP carrier protein, mitochondrial	F:catalytic activity; C:membrane	no IPS match
S08_106280443	8	106280443	0.33	3.85E-11	Agamous-like MADS-box protein AGL16	F:DNA-binding transcription factor activity; C:nucleus; P:regulation of transcription, DNA-templated	no IPS match
S08_105353008	8	105353008	0.29	2.54E-10	Putative retrotransposon protein, identical	F:carboxypeptidase activity; F:binding; P:nitrogen compound metabolic process; P:macromolecule metabolic process; P:primary metabolic process	no IPS match
S08_106308670	8	106308670	0.31	3.35E-09	[Pyruvate dehydrogenase (acetyl-transferring)] kinase, mitochondrial	F:binding	no IPS match
S08_106726114	8	106726114	0.27	2.10E-08	PREDICTED: uncharacterized protein LOC107865331	F:nucleic acid binding; P:DNA integration	no IPS match
S08_105142620	8	105142620	0.44	3.22E-07	PREDICTED: uncharacterized protein LOC107861928	F:zinc ion binding	no IPS match
S08_105183429	8	105183429	0.29	4.79E-07	keratin, type I cytoskeletal 12-like	F:protein tyrosine phosphatase activity; F:binding; P:proteolysis; P:transport; F:protein tyrosine/serine/threonine phosphatase activity; F:cysteine-type peptidase activity; C:integral component of membrane; P:peptidyl-tyrosine dephosphorylation; C:cytoplasmic part; C:membrane protein complex	no IPS match
S08_106609467	8	106609467	0.32	5.13E-07	Putative polyprotein, identical	F:nucleic acid binding; F:zinc ion binding; P:DNA integration	no IPS match
S10_226847725	10	226847725	0.46	1.05E-06	hypothetical protein CQW23_01337	F:copper ion binding; F:oxidoreductase activity; P:oxidation-reduction process	no IPS match
S11_244407143	11	244407143	0.19	7.57E-08	PREDICTED: uncharacterized protein LOC104231117		no IPS match

## Discussion

The greater the genetic diversity of germplasm, the greater is the chance of success in breeding desirable strains. Knowledge of population structure and genetic diversity is essential for association mapping studies, genomic selection and the classification of individual genotypes into different groups. In the present study, we classified Ethiopian *Capsicum* germplasms into different species and analyzed their genetic diversity. According to our classification, the majority of Ethiopian pepper germplasms collected from diverse agro-ecologies are *C*. *annuum*, whose mature fruit is an integral ingredient of the local spice mixture called *berbere*, used to season many Ethiopian dishes. The green fruit of *C*. *annuum* is also a very important component in the daily diet. The brown chilli pepper type (*C*. *annuum*) is especially highly valued for its high pungency for flavoring and coloring. Work by Berhanu et al. [47], Shimelis et al. [12] and Abrham et al. [13] had demonstrated the prevalence and variability of *C*. *annuum*. We also recognized the presence of some *C*. *frutescens* collections, known locally as "mitmita", growing on some part of the country. They are known locally for being highly pungent. Yayeh (1998) had previously described the existence of *C*. *frutescens* in Ethiopia [11]. In addition, the distribution of these two *Capsicum* species across Africa was described by Eshbaugh (1983), who summarized the evolutionary history of peppers and described how the genus was introduced to East Africa [48]. Similarly, Dagnoko et al. (2013) mentioned the importance of *C*. *annuum* and *C*. *frutescens* in West African countries [49].

Although morphological traits are important in the study of genetic diversity, because of their mostly polygenic nature and their dependence on various environmental factors, they may not always reflect real genetic variation [50]. Owing to their ability to recognize specific DNA sequences in the closely related genotypes, SNP markers have been used successfully to estimate genetic diversity among different plants. GBS is a preferred high-throughput genotyping method involving targeted complexity reduction and multiplex sequencing to produce high-quality polymorphism data at a relatively low cost per sample [35]. The GBS method uses restriction enzymes coupled with DNA-barcoded adapters and can simultaneously perform SNP discovery and genotyping with or without reference genome sequences. GBS have been applied to various approaches for plant breeding and plant genetic studies, including linkage maps [20, 36, 51], genome-wide association studies [20, 28], genomic selection [52] and genomic diversity studies [7].

We performed GBS for genotyping 142 germplasms of *Capsicum* species. Two enzymes, *Pst*I and *Mse*I, were used to reduce genome complexity, consistent with previous studies by Han et al. [20]. Using the CM334 genomic reference, SNP calling generated 53,284 high-quality SNPs. Transitions (72.45%) were more frequent than transversions (27.55%). The percentages of each SNP type in our study were 36.08%, 36.37%, 8.66%, 5.49%, 8.49% and 4.91% for [AG], [CT], [GT], [AT], [AC] and [CG], respectively. This observation agrees with the report of Taranto et al. [4], which also found a higher frequency of transitions than transversions.

Heritability values are helpful in predicting the expected progress to be achieved through the process of selection; high heritability coupled with high genetic advance (GA) is an indicator of a high proportion of additivity in the genetic variance, and consequently suggests that a high genetic gain can be expected from selection [31]. The heritability values of the traits assessed in this analysis fell into two categories—very high (98.1%) for fruit weight and moderately high for the remainder—as illustrated in previous studies [9, 31]. High GA (>20%) with moderate heritability values were observed for pericarp thickness, fruit width, pedicel length and fruit length. This pattern is partly supported by the findings of Usman et al. [53]. High values of PCV and GCV values for some of fruit-related traits were also reported previously [12] and indicated the existence of substantial variability, ensuring ample scope for improvement of these traits through selection.

Our panel of germplasms exhibited a wide range of genetic diversity for different agro-morphological traits. Plant habits as a measure of plant architecture; growth parameters including plant height, stem thickness, internode length, number of side branches; and morphological traits such as number of flowers per axil, length and thickness of fruits, fruit weight and number of locules all showed variation among germplasms.

Results of neighbor-joining clustering with model-based STRUCTURE, phylogenetic and PCoA all similarly suggested that there are two genetically distinct subclasses among the Ethiopian *Capsicum* germplasms investigated in this study. The PCoA showed tight clustering within the first clade, composed of *C*. *annuum*, and the second clade, in which all *C*. *frutescens* are grouped. A previous diversity study of 39 cultivated Ethiopian *C*. *annuum* strains using AFLP distance estimation, however, showed four major clusters [3]. Although *C*. *frutescens* has been shown to have close affinity to *C*. *annuum*, they were grouped separately in this study [5]. Based on our data, the high diversity values in the two subpopulations suggests the existence of excessive genetic variation within them. The lower value of *H*_o_ (observed heterozygosity) as compared to *H*_e_ (expected heterozygosity) in the subpopulations indicated the presence of inbreeding in the majority of Ethiopian *Capsicum* germplasms. The average distance (*H*_E_) between individuals in same clade value was lower in subpopulation C1 (0.05) than in subpopulation C2 (0.07), indicating that C1 contained less variation. Genetic differentiation (*F*_ST_) value for subpopulations C1 and C2 were 0.713 and 0.85, respectively, predicting that the germplasms in the two clades have several genotype patterns. There was a significant correlation between some of the morphological traits, such as plant height and fruit width, as indicated by SNP-marker-based matrices (S7 Table). In summary, the model-based ancestry analysis, the phylogenetic tree and the PCoA strongly supported the possibility that the collection of Ethiopian *Capsicum* germplasms has two well-differentiated genetic populations and some admixtures.

In our research, additional information was provided by the GWAS analysis. As was shown earlier by Chaim et al. (2001) [54] and Han et al. (2016) [51], the highest numbers of SNP markers for various agronomic traits were detected on chromosome 3. From the total of 398 significant SNPs of selected traits (S8 Table), 10 SNPs from chromosome 8, 6 SNPs from chromosome 7 and 1 SNP from chromosome 3 were common for the traits of fruit weight and fruit length. The remaining major SNP marker (10) observed for fruit weight in our study was detected by Chaim et al. [54]. A recent report by Chunthawodtiporn et al. [55] demonstrated the distribution of fruit-trait QTL; the authors considered transverse and longitudinal section, fruit shape and blossom-end shape on all chromosomes except 4, 5 and 7. However, our study included more fruit-related traits, and our results suggest that significant SNP markers are found on all chromosomes. For leaf length, for which we identified various significant SNP markers on chromosomes 2, 3, 6, 7 and 9, co-localized QTL results were reported on chromosomes 6, 8, 9 and 11 by Han et al. [51] and on chromosomes 1, 2 and 3 by Chunthawodtiporn et al. [55]. We detected SNP markers for stem traits on seven of the same chromosomes reported by Han et al. [51].

GWAS identified 31 SNP markers for fruit weight in this study. On chromosome 3, the SNP markers were co-localized at two different locations with the QTL reports of Han et al. (2016), Chaim et al. (2001) and Nimmakayala et al. (2016) [28, 51, 54], at 126 Mbps and 222 Mbps. In a pattern similar to that of our results, Nimmakayala et al. (2016) reported a QTL region between 220 and 237 Mbps on chromosome 10. Although both that study and Chaim et al. (2001) reported QTL positions on chromosome 8, our results for chromosome 8 indicated the presence of a SNP concentration at a different location. Similarly, the SNP position we identified on chromosome 11 at 239 Mbp is different from that reported by Nimmakayala et al. (2016) of a QTL elsewhere on the same chromosome.

This study is the first detailed characterization of a large sample of the Ethiopian *Capsicum* germplasms, representing the six administrative regions covering all *Capsicum*-growing agro-ecologies in the country. Our morphological and molecular characterization provides insight into the genetic variability of *Capsicum* in Ethiopia.

## Conclusions

The phenotypic and molecular characterization of Ethiopian *Capsicum* germplasms showed high variation that can be exploited for the further breeding. Two distinct subpopulations were identified from the 130 accessions used in this study based on SNP information from our GBS library. Model-based population structure, phylogenic study and PCoA revealed similar results. Probably because of the existence of a dominant informal seed-distribution system in the country, the majority of germplasms collected from different geographical areas were grouped into the same or different clades, without clear association with growing region. However, clade 1, which contained the majority of germplasms, consisted mainly of *C*. *annuum* and *C*. *baccatum*. Clade 2 was composed mainly of *C*. *frutescen*s accessions collected from different growing regions of the SNNPs region. GWAS analysis helped us to further identify significant markers associated with important morphological traits, some of which were co-localized with reported QTLs. Besides providing additional information to identify candidate genes for the traits considered, this study reconfirms the validity of using GBS and downstream analysis for marker-assisted selection in *Capsicum*.

## Supporting information

S1 Table Morphological data from 142 *Capsicum* germplasms and KMO/Bartlett's test analysis results. (XLSX)

S2 Table GBS library construction. (XLSX)

S3 Table *Q* matrix of population structure. (XLSX)

S4 Table Gene diversity data. (XLSX)

S5 Table HRM markers used for species identification. (DOCX)

S6 Table Distribution of SNPs across the 12 chromosomes. (DOCX)

S7 Table Summary of agronomic and morphological traits exhibiting high correlation with the estimated ancestry membership coefficients (*Q*). (DOCX)

S8 Table Significant SNP information of GWAS result for 11 traits. (XLSX)

S1 Fig Flower morphology showing variation among species. (DOCX)

S2 Fig HRM melting curves. (DOCX)

S3 Fig Box plot of selected phenotypes. (DOCX)

S4 Fig Significant SNP marker distribution for fruit-, leaf- and stem-related traits based on GWAS result. (DOCX)
