# Supplementary figures and images for: Genetic diversity and population structure of Ethiopian Capsicum germplasms
Source: PLoS One. 2019 May 21;14(5):e0216886. doi: 10.1371/journal.pone.0216886 (PMC6528999; doi:10.1371/journal.pone.0216886)

**S5 Table.** HRM markers used for species identification (Jeong et al, 2010)


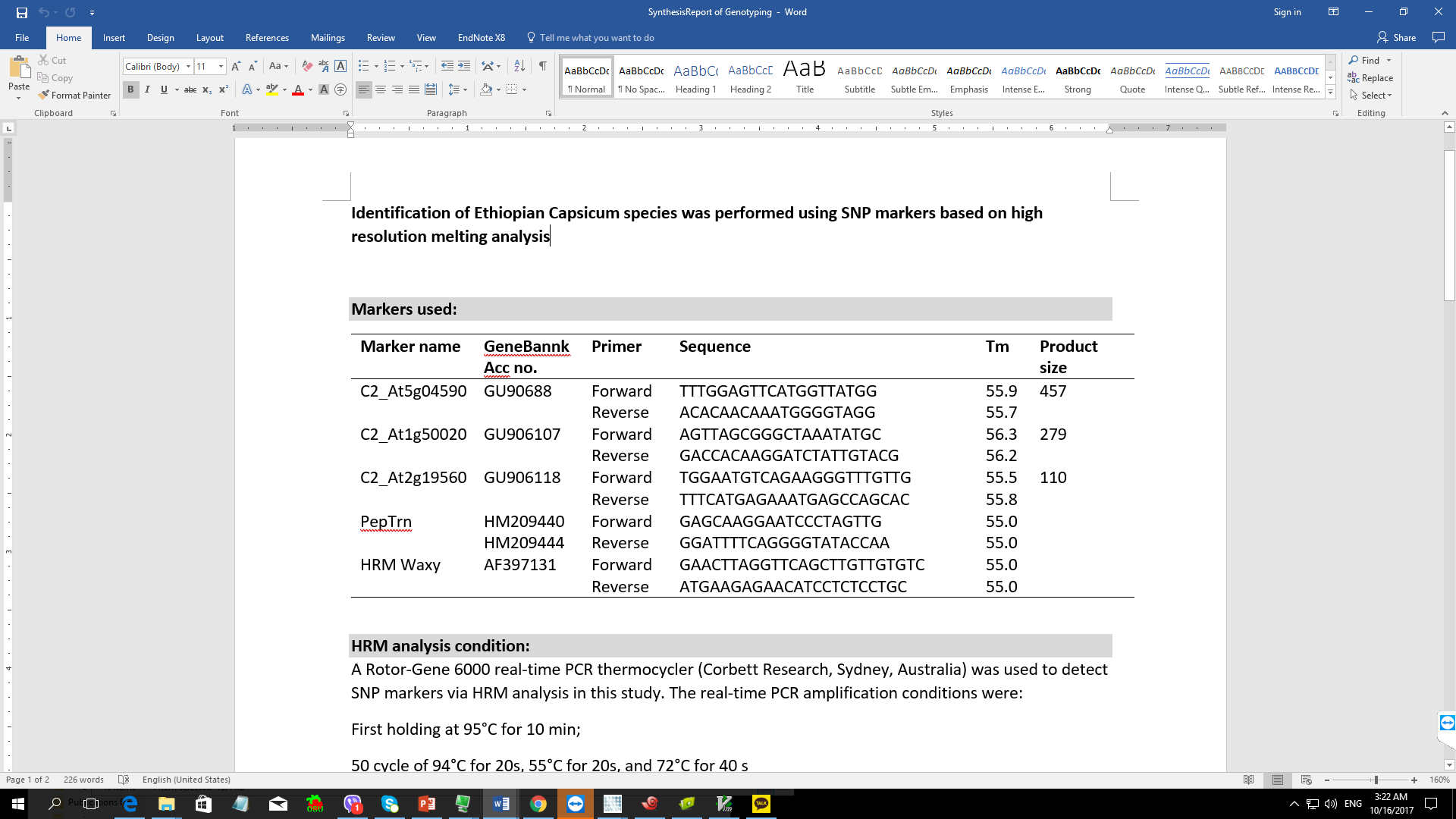

Supplement: S5 Table — (DOCX) [file pone.0216886.s005.docx]

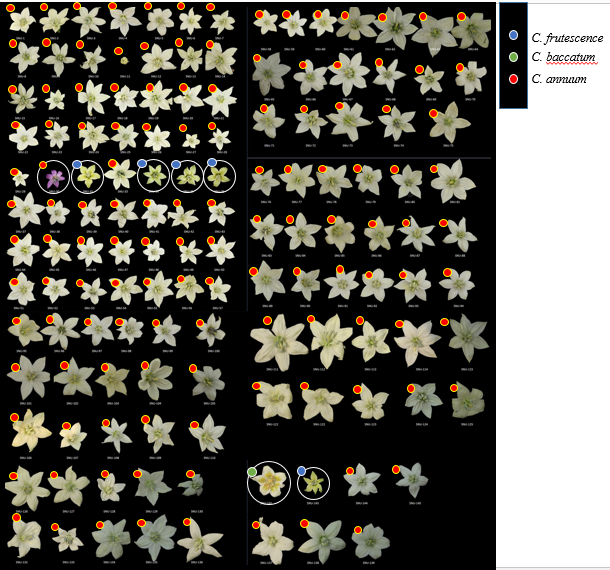


**S1 Fig**. Flower morphology of Ethiopian Capsicum

Supplement: S1 Fig — (DOCX) [file pone.0216886.s009.docx]

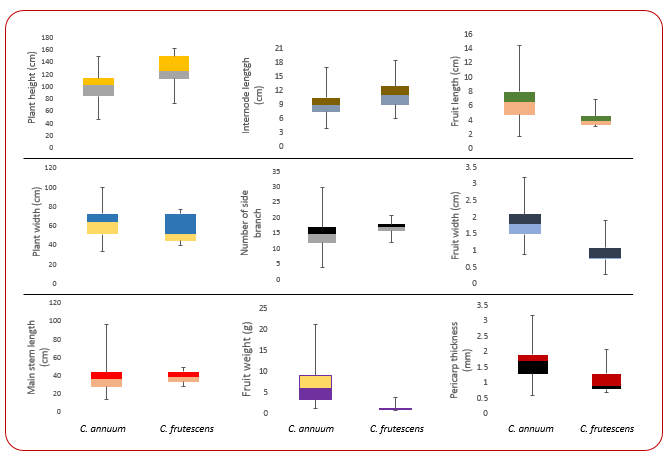


**S3 Fig**. Box plot of selected nine phenotypes of *Capsicum annuum* and *Capsicum frutescens*

Supplement: S3 Fig — (DOCX) [file pone.0216886.s011.docx]
