# Supplementary material for: Genetic diversity and population structure of Ethiopian Capsicum germplasms
Source: PLoS One. 2019 May 21;14(5):e0216886. doi: 10.1371/journal.pone.0216886 (PMC6528999; doi:10.1371/journal.pone.0216886)
Supplement: S6 Table — (DOCX) [file pone.0216886.s006.docx]

**S6 Table.** The chromosomal distribution and proportion of polymorphic markers used for computing heterogeneity, genetic distance and principal coordinate analysis and population structure

| Chromosome | No. of markers | Proportion |
| --- | --- | --- |
| 1 | 1677 | 12.0% |
| 2 | 974 | 7.0% |
| 3 | 1475 | 10.5% |
| 4 | 1179 | 8.4% |
| 5 | 1104 | 7.9% |
| 6 | 1150 | 8.2% |
| 7 | 1166 | 8.3% |
| 8 | 712 | 5.1% |
| 9 | 1127 | 8.1% |
| 10 | 928 | 6.6% |
| 11 | 1232 | 8.8% |
| 12 | 1274 | 9.1% |
| Total | 13998 | 100.0% |
