# Supplementary material for: Genetic diversity and population structure of Ethiopian Capsicum germplasms
Source: PLoS One. 2019 May 21;14(5):e0216886. doi: 10.1371/journal.pone.0216886 (PMC6528999; doi:10.1371/journal.pone.0216886)
Supplement: S7 Table — (DOCX) [file pone.0216886.s007.docx]

**S7 Table**. Summary of agronomic and morphological traits exhibiting high correlation with the estimated ancestry membership coefficients (Q) in the STRUCTURE software

| Species | Number of Accession | Plant height (cm) | Internode length (cm) | Fruit length (cm) | Fruit width | Corolla color | Stem color |
| --- | --- | --- | --- | --- | --- | --- | --- |
| *C. annuum* | 121 | 48-175 | 4-17 | 1.8-14.4 | 0.9-3.2 | White, yellowish and purple | Green, green with purple stripe & with purple dot; and purple |
| *C. frutescens* | 9 | 74-163 | 6-18.5 | 3.2-6.9 | 0.3-1.9 | White and greenish yellow | Green and green with purple stripe |
| *C. baccatum* | 1 | 175 | 13 | 7.7 | 1.5 | White with yellow spots in the center | Green |
