# Supplementary material for: Genetic diversity and population structure of Ethiopian Capsicum germplasms
Source: PLoS One. 2019 May 21;14(5):e0216886. doi: 10.1371/journal.pone.0216886 (PMC6528999; doi:10.1371/journal.pone.0216886)
Supplement: S2 Fig — (DOCX) [file pone.0216886.s010.docx]

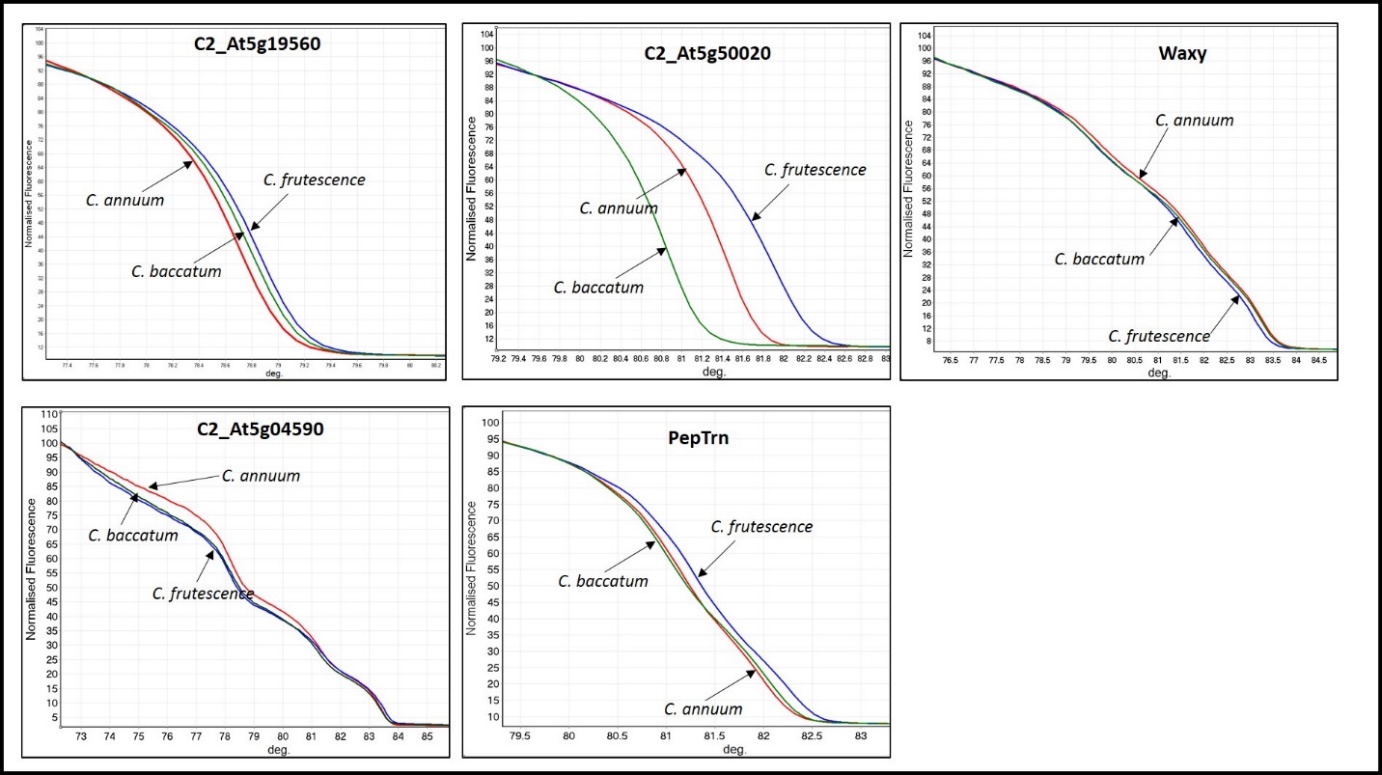


**S2 Fig**. Melting curve analysis (HRM) developed from COSII. The COSII markers used are: (a) C2_At5g1956, (b) C2_At5g50020, (c) C2_At5g04590, (d) Waxy and (e) PepTrn.
