# Supplementary material for: Genetic diversity and population structure of Ethiopian Capsicum germplasms
Source: PLoS One. 2019 May 21;14(5):e0216886. doi: 10.1371/journal.pone.0216886 (PMC6528999; doi:10.1371/journal.pone.0216886)
Supplement: S4 Fig — (DOCX) [file pone.0216886.s012.docx]

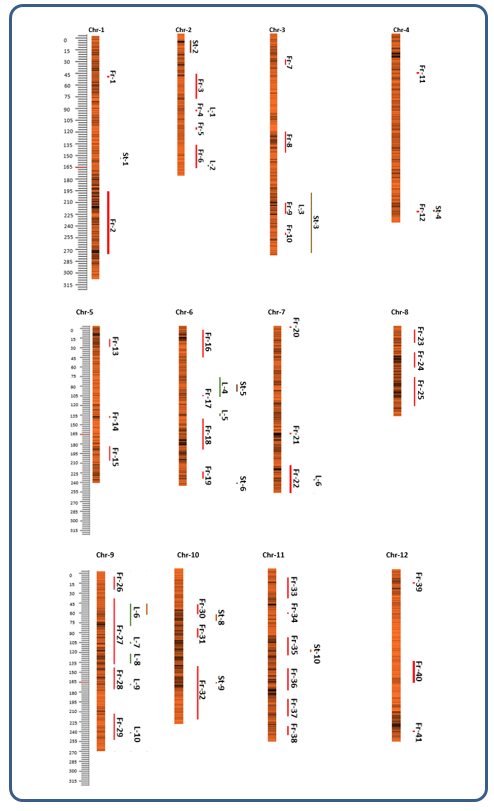


**S4 Fig**. Significant SNP marker distribution map across 12 chromosomes of *Capsicum* for fruit, leaf and stem traits based on GWAS result.
